# Determining HER2 Status by Artificial Intelligence: An Investigation of Primary, Metastatic, and HER2 Low Breast Tumors

**DOI:** 10.3390/diagnostics13010168

**Published:** 2023-01-03

**Authors:** Christiane Palm, Catherine E. Connolly, Regina Masser, Barbara Padberg Sgier, Eva Karamitopoulou, Quentin Simon, Beata Bode, Marianne Tinguely

**Affiliations:** 1 Pathologie Institute Enge, 8005 Zurich, Switzerland; 2 Faculty of Medicine, University of Zurich, 8006 Zurich, Switzerland

**Keywords:** HER2 immunohistochemistry, HER2 in situ hybridization, artificial intelligence, digital pathology, breast carcinoma

## Abstract

The expression of human epidermal growth factor receptor 2 (HER2) protein or gene transcripts is critical for therapeutic decision making in breast cancer. We examined the performance of a digitalized and artificial intelligence (AI)-assisted workflow for HER2 status determination in accordance with the American Society of Clinical Oncology (ASCO)/College of Pathologists (CAP) guidelines. Our preliminary cohort consisted of 495 primary breast carcinomas, and our study cohort included 67 primary breast carcinomas and 30 metastatic deposits, which were evaluated for HER2 status by immunohistochemistry (IHC) and in situ hybridization (ISH). Three practicing breast pathologists independently assessed and scored slides, building the ground truth. Following a washout period, pathologists were provided with the results of the AI digital image analysis (DIA) and asked to reassess the slides. Both rounds of assessment from the pathologists were compared to the AI results and ground truth for each slide. We observed an overall HER2 positivity rate of 15% in our study cohort. *Moderate* agreement (Cohen’s κ 0.59) was observed between the ground truth and AI on IHC, with most discrepancies occurring between 0 and 1+ scores. Inter-observer agreement amongst pathologists was *substantial* (Fleiss´ κ 0.77) and pathologists’ agreement with AI scores was 80.6%. *Substantial* agreement of the AI with the ground truth (Cohen´s κ 0.80) was detected on ISH-stained slides, and the accuracy of AI was similar for the primary and metastatic tumors. We demonstrated the feasibility of a combined HER2 IHC and ISH AI workflow, with a Cohen’s κ of 0.94 when assessed in accordance with the ASCO/CAP recommendations.

## 1. Introduction

Approximately 15–20% of newly diagnosed invasive breast cancers (IBC) overexpress the human epidermal growth factor receptor 2 (HER2) oncogene, which is associated with increased tumor progression and metastasis [1,2,3]. Since HER2 positive tumors can be targeted with medical therapies, a reliable method is necessary to determine the HER2 status on both primary and metastatic breast tumors [4,5]. The standard diagnostic workflow involves immunohistochemistry (IHC) and in situ hybridization (ISH) methods with manual assessment by pathologists. According to the 2018 American Society of Clinical Oncology (ASCO)/College of American Pathologists (CAP) guidelines, both completeness and intensity of HER2 membrane staining must be evaluated to determine the HER2 status on IHC-stained slides [6]. However, the visual assessment of stained slides is subject to inter-pathologist variability, and unusual or heterogeneous staining patterns often present diagnostic challenges [7,8]. Therefore, pathologists may strategically assess relatively more cases as score 2+, in order to defer them for ISH testing, which, although more conclusive in determining HER2 positive and negative cases in the IHC 2+ cohort, is significantly more cost- and labor-intensive [9].

Recent and emerging evidence provides a strong case for moving away from the dichotomous ‘positive’ or ‘negative’ reporting of HER2 status, in favor of identifying a category of IBC with a low HER2 status as they demonstrate a response to new anti-HER2 antibody-conjugate therapy. According to the recently published DESTINY-Breast 04 trial results, HER2 low tumors were defined as a score of 1+ on IHC or 2+ on IHC with a negative ISH result, and this cohort of patients demonstrated significantly longer progression-free and overall survival when treated with trastuzumab deruxtecan in comparison to chemotherapy [10]. Currently, the DAISY trial is now underway and seeks to further investigate the efficacy of this drug in three cohorts: HER2 overexpressing (HER2 IHC 3+ or IHC 2+ with positive ISH) vs. HER2 low-expressing (IHC 1+ or IHC 2+ with negative ISH) vs. HER2 non-expressing (IHC 0) [11]. Therefore, the ability of pathologists to accurately classify HER2 IHC scores and interpret ISH in a standardized manner, with minimal inter-observer variability, is of upmost priority.

Technological advances, specifically in the field of artificial intelligence (AI) and digital image analysis (DIA), offer the added potential to minimize inter- and intra-pathologist variability in the scoring process by offering an objective comparative value [12,13,14,15,16,17,18,19]. Several studies have analyzed the potential role of AI in determining the HER2 status and DIA has been acknowledged as a possible diagnostic modality in the current ASCO/CAP guidelines. However, to our knowledge, no research has been published on the AI-assisted assessment of HER2 status, incorporating both the IHC and ISH methods into a complete workflow. Since both IHC and ISH supplement each other in the diagnostic process of determining HER2 status, and are described in this way in the ASCO/CAP guidelines, we aimed to investigate whether an approach that incorporates algorithms for both methods could offer the chance of a more effective and objective diagnosis [6]. Additionally, no studies were identified in the literature that assesses the performance of AI on metastatic or HER2 low breast tumors. Therefore, we sought to evaluate a novel AI-assisted workflow including both IHC and ISH for determining the HER2 status of primary and metastatic breast cancer.

## 2. Materials and Methods

### 2.1. Setting and Ethics

This study was conducted at Pathologie Institut Enge AG, Switzerland, and was approved by the Ethics Committee of the Canton of Zurich (BASEC-Nr.: 2021-00210).

### 2.2. Cohort Selection, Tissue Staining and Interpretation

**Figure 1:**
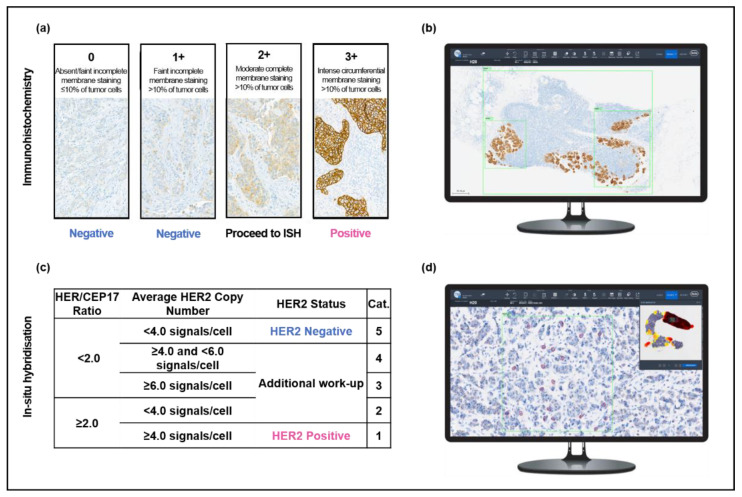
Overview of the study protocol for HER2 evaluation. Pathologists performed manual, light microscopic evaluation of HER2 (**a**) immunohistochemistry and (**c**) in situ hybridization in accordance with the 2018 ASCO/CAP guidelines. Slides were also analyzed with the support of two AI algorithms: AI-assisted immunohistochemistry analysis (**b**) was performed by placing three regions of interest (green) over tumor tissue and (**d**) AI-assisted in situ hybridization was analyzed by selecting a region of interest (green) within an area of high HER2 expression, as indicated by the heatmap (upper right). Within the region of interest, the algorithm calculated the HER2 and CEP17 signals for 20 cells (red).

Core needle biopsies (CNB) of 495 newly diagnosed primary IBCs (category B5b) and 30 metastatic breast carcinomas consecutively diagnosed at our institute throughout 2020 were identified using the institutional lab informatics system (PathoWin+, Basys Data, Basel, Switzerland). Existing IHC slides for all primary breast carcinoma cases in 2020 were retrieved, digitalized, and analyzed (see the method in Section 2.3) as a preliminary cohort. For further analyses, we included all 30 metastatic tumors and their matched primaries and a further random selection of primary tumors from the preliminary cohort (total study cohort, *n* = 97). The 2 µm (IHC) and 4 µm (ISH) sections were newly cut from routinely processed formalin-fixed paraffin embedded tissues and mounted on TOMO Adhesion Microscope Slides (Matsunami Glass, Japan). Both IHC and ISH were performed using the Ventana BenchMark ULTRA automated slide stainer with the Ultraview Detection Kit (Roche Diagnostic International, Rotkreuz, Switzerland). IHC and ISH were performed in accordance with the vendor’s package insert protocols using the VENTANA anti-HER2/neu (4B5) rabbit monoclonal primary antibody and VENTANA HER2 Dual ISH DNA Probe Cocktail Assay, respectively (Roche Diagnostic International, Rotkreuz, Switzerland). ISH was performed on 55/97 samples, corresponding to all cases with an IHC HER2 score of ≥1+. Both IHC and ISH were interpreted according to the 2018 ASCO/CAP guidelines and HER2 low tumors were defined as IHC 1+ or IHC 2+ with negative ISH (Figure 1a) [6,10].

### 2.3. AI Analysis of Immunohistochemistry Slides

IHC slides were digitalized using a VENTANA DP200 (Roche Diagnostic International, Rotkreuz, Switzerland) and analyzed using the HER2 4B5 algorithm in the uPath enterprise software (Roche Diagnostic International, Rotkreuz, Switzerland). Three regions of interest (ROI) were selected per slide: two smaller ROIs were placed over separate areas with at least 100 tumor cells (“area 1” and “area 2”), and a large ROI was placed over all tumor tissue on the slide (“area 3”). The mean score of these three areas was calculated and taken to represent the AI-determined HER2 IHC score (Figure 1b). This method was developed in order to minimize ROI- and user-dependent factors in the AI evaluation.

### 2.4. AI Analysis of In Situ Hybridization Slides

ISH slides were digitalized using the VENTANA DP200 and analyzed using the HER2 Dual ISH image analysis algorithm (Roche Diagnostic International, Rotkreuz, Switzerland). The algorithm enables the selection of one ROI, which is aided by a heatmap overlay highlighting areas of the slide with the highest HER2 expression (Figure 1d). Within the selected ROI, the algorithm identifies 20 cells with the highest HER2 amplification, provides HER2 and CEP17 counts for each cell, and calculates the ratio. As our aim was to understand the performance of an AI image analysis protocol for HER2 status determination, the manual correction features in both the IHC and ISH algorithms were not used.

### 2.5. Manual Assessment of Slides

To compare the pathologists´ assessments to the AI results, three pathologists were asked to independently assess the IHC slides by light microscopy as they would in routine diagnostics (Group: “Pathologists”). Following a minimum washout period of 2 weeks, they were provided with the AI results and asked to re-evaluate the IHC slides per microscope (Group: “AI-assisted Pathologists”). The ground truth for the IHC results was defined as the consensus score reached by the three pathologists for each case. The ground truth for the ISH results was determined by a single pathologist counting the HER2 signals per cell and the HER2/CEP17 ratio for 20 cells. Area selection was performed following a review of the ISH slide and paired IHC slide to determine potential areas of HER2 amplification. For equivocal results, a second pathologist recounted the ISH signals of 20 cells, and diagnoses were allocated in accordance with the 2018 ASCO/CAP guidelines [6].

### 2.6. Statistical Analysis

To assess the accuracy and compare the assessments by AI and pathologists to our defined ground truth, we calculated the Cohen’s κ. Inter-observer variability was measured with Fleiss’ κ. The interpretation of agreement was performed according to Landis and Koch: κ-values 0.01–0.20 = slight agreement, 0.21–0.40 = fair agreement, 0.41–0.60 = moderate agreement, 0.61–0.8 = substantial agreement, 0.81–1.0 = almost perfect agreement [20]. The sensitivity and specificity values were calculated for each diagnostic step in HER2 status determination including the overall workflow and our level of significance was set at *p* < 0.05. To assess the significance of difference in kappa between observations, two-sided pairwise t-tests were performed. The Pearson Chi2 test was used to compare the agreement levels and their significance. Statistical calculations were performed using IBM software SPSS Version 27 and Microsoft Excel.

## 3. Results

### 3.1. Clinicopathological Features

During 2020, there were 495 cases of B5b IBC diagnosed at our institute, and all cases were included in our preliminary cohort (Table 1). The average age of patients was 61 years (range 29–95 years), and all but two were women. The most prevalent tumor type was invasive carcinoma of no special type (80%) and our estrogen receptor (ER), progesterone receptor (PR), and HER2 positivity rates were 85%, 75%, and 12%, respectively. The distribution of tumor grading included: 23% grade 1, 48% grade 2, and 29% grade 3. There were 97 CNBs (67 primary breast tumors, 30 metastases) included in our study cohort for further analyses. All tissue samples of this cohort were from women and the mean age of the study cohort was 60 years (range 30–80 years). The majority of primary tumors were invasive carcinoma of no special type (82%), and most tumors were classified as grade 2 (45%), followed by grade 3 (42%) and grade 1 (14%). ER was positive in 87% and PR in 78%. The reported HER2 positivity rate was 15%. At the time of original diagnosis, 20 tumors were investigated with ISH for HER2 status determination in addition to routine IHC. Metastatic sites included lymph nodes (*n* = 22) and liver (*n* = 8), and the mean size of the metastatic lymph node deposits was 4.99 mm (range 0.3–12 mm).

### 3.2. Preliminary Cohort

From our preliminary cohort of 495 cases of B5b primary invasive breast carcinoma, we retrieved a total of 475 IHC slides with adequate tissue and staining for digitalization and analysis. The remaining 20 slides were excluded as they were not suitable for further analysis. The IHC scoring distribution according to manual assessment by pathologists included 181 cases as IHC score 0 (38.1%), 156 cases as 1+ (32.8%), 87 cases as 2+ (18.3%), and 51 cases as 3+ (10.7%). In contrast, the AI-IHC algorithm identified 22 cases as 0 (4.6%), 137 cases as 1+ (28.8%), 254 cases as 2+ (53.5%), and 62 cases as 3+ (13.1%). In total, we observed only 182/475 concordant cases (38.3%) between the AI and pathologists, and the AI overestimated the number of 2+ cases in comparison to the pathologists (254 cases vs. 87 cases). In 184/475 cases (38.7%), the discordance observed in scoring would lead to significant consequences such as the inclusion or omission of HER2 ISH testing, or a change in the tumors’ HER2 status. Following analysis of our preliminary cohort and discussions with the vendor, we identified deviations in our round robin tested laboratory HER2 IHC staining protocol in comparison with the vendor’s recommended protocol for use with the AI software. Consequently, we made changes to our total Ultra Cell Conditioning Solution (CC1) incubation time (172 min to 56 min), antibody incubation time (24 min to 12 min), and counterstaining time (8 min to 4 min) to conform to the vendor’s recommendations. New sections were prepared from the FFPE tissue for our study cohort (*n* = 97) and were stained according to the adjusted protocol, with the results shown in Section 3.3, Section 3.4, Section 3.5, Section 3.6 and Section 3.7. We observed an overall improvement in concordance between the AI and manual assessment in our study cohort primary tumors (*n* = 67), with 77.6% of cases showing full concordance, and only 4.5% showing discordance with diagnostic consequence. There were 57 cases that were part of both our preliminary and study cohorts and the comparative results using our laboratory and amended staining protocols are presented in Supplementary Table S1.

### 3.3. Primary B5b IBC: Immunohistochemistry

**Figure 2:**
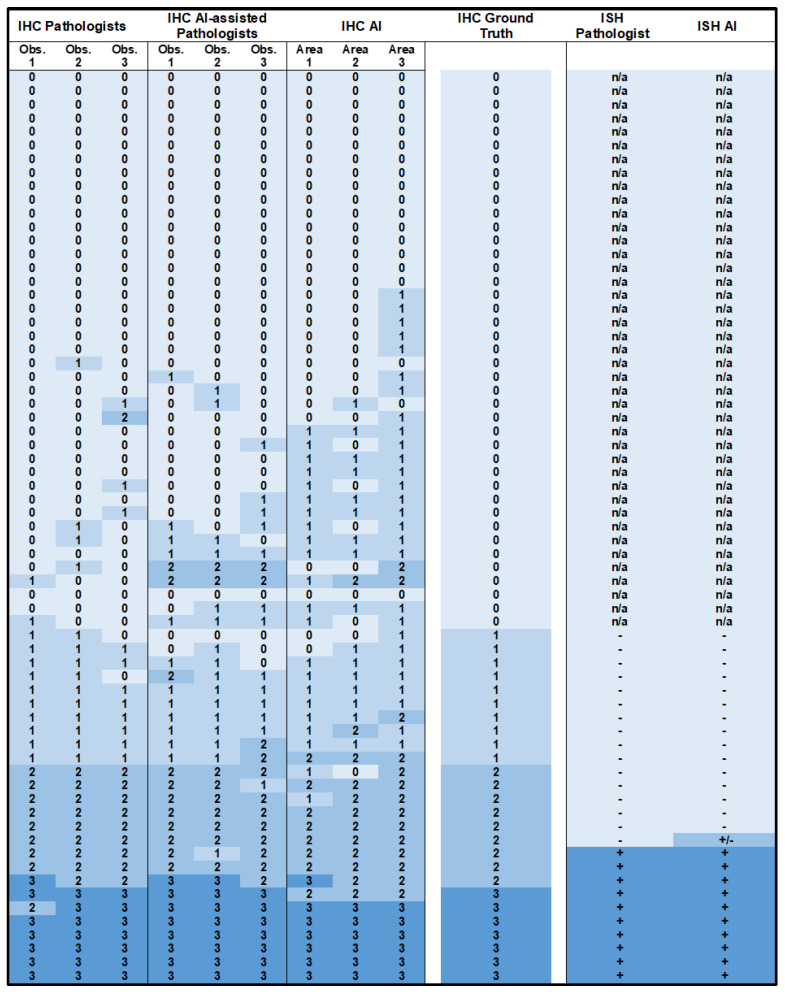
Heatmap representation of HER2 scoring for primary B5b invasive breast carcinomas. IHC scores (0, 1, 2, 3) are shown for Pathologists, AI-assisted Pathologists, and AI in comparison to the ground truth. ISH was designated as negative (−), positive (+), or requiring further assessment (+/−).

In our study cohort, the AI-IHC algorithm had moderate concordance with the ground truth (Cohen’s κ 0.59, 95% CI 0.43–0.75). When pathologists were asked to score the IHC slides with the assistance of AI, the “AI-assisted Pathologists” concordance with the ground truth was almost perfect (Cohen’s κ 0.89, 95% CI 0.77–1.0), with individual concordance rates of Cohen’s κ 0.71, 0.71, and 0.61. The greatest discrepancies in scoring between “Pathologists” and AI occurred on the IHC slides with a ground truth score of 0 (Figure 2). When the “Pathologists” were provided with the AI results, their overall agreement with the algorithm’s findings increased by 9% (71.6% vs. 80.6%), indicating that pathologists were likely to adjust their assessment to match that of the algorithm.

**Figure 3:**
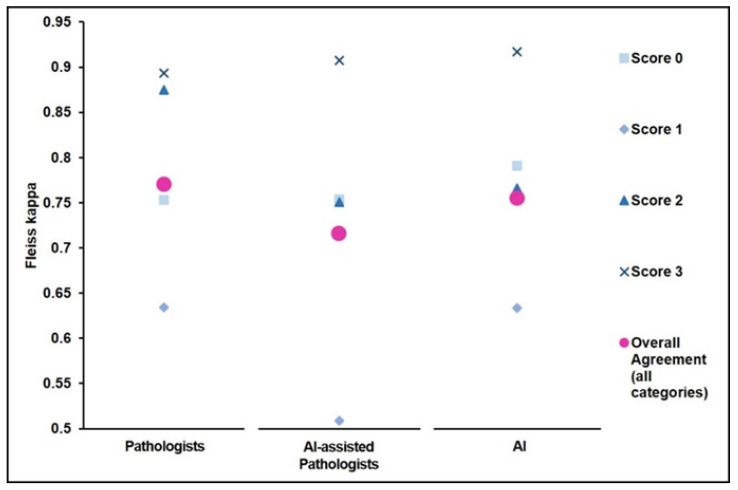
Evaluation of inter-observer agreement. Fleiss’ kappa for the measurement of inter-observer agreement amongst Pathologists, AI-assisted Pathologists, and AI on the IHC-stained slides. Agreement is presented for each IHC score and combined.

We observed substantial inter-observer agreement in the assessment of the IHC slides within the groups “Pathologists” (Fleiss κ 0.77, 95% CI 0.68–0.86) and “AI-assisted Pathologists” (Fleiss κ 0.74, 95% CI 0.65–0.82). As shown in Figure 3, inter-observer agreement was highest for cases rated as score 3+ (Fleiss κ 0.89, 95% CI 0.75–1.0) and lowest for cases assessed as 1+ (Fleiss κ 0.63, 95% CI 0.50–0.77).

### 3.4. Primary B5b IBC: In Situ Hybridization

**Figure 4:**
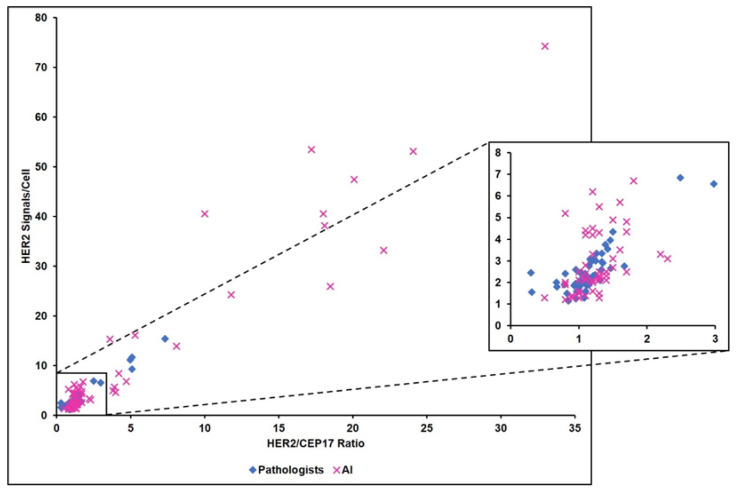
Comparison of the calculated HER2/CEP17 ratio and the HER2 signals/cell between Pathologists and AI-assisted DIA on the ISH-stained slides.

A total of 26/67 cases, corresponding to all cases with an IHC HER2 consensus score of 1+ or above, were evaluated by ISH. Concordant results were noted between AI and the ground truth HER2 status in 25/26 cases, and AI demonstrated almost perfect accuracy (Cohen’s κ 0.92, 95% CI 0.77–1.0). The AI-ISH algorithm classified eight cases into categories 2, 3, or 4 (Figure 1c). As per the ASCO/CAP guidelines, seven of these would be considered negative following the inclusion of their IHC 1+ scores, and one would require a second observer to count 20 cells on the ISH slide, as the IHC score was 2+. AI-recounts were not performed, therefore, our discordant case is depicted as +/− in Figure 2. Notably, the AI-ISH algorithm occasionally assigned unexpectedly high HER2 counts of up to 74 signs/cell and HER2/CEP17 ratios of up to 15, and on average, the AI counts amounted to over three times that from the Pathologists (Figure 4). However, the tendency of AI to provide exaggerated counts did not significantly affect the outcomes, with only one negative case being upgraded by the AI, and as per the ASCO/CAP guidelines, this case would require reassessment by ISH.

### 3.5. Primary B5b IBC: HER2 Low Tumors

According to our defined ground truth, the study cohort contained 16 HER2 low tumors. For this group, the level of concordance between the AI and the ground truth was moderate (Cohen’s κ 0.54, 95% CI 0.19–0.90). However, the AI classified 20 tumors as IHC 1+ and seven tumors as IHC 2+/ISH negative, totaling 27 HER2 low tumors and increasing our proportion of HER2 low tumors by 16%. For our ground truth HER2 low tumors, we observed substantial inter-observer agreement in classification amongst Pathologists (Fleiss κ 0.78, 95% CI 0.55–1.0), which was slightly lower, but remained substantial, in the “AI-assisted Pathologists” group (Fleiss κ 0.61, 95% CI 0.40–0.81).

### 3.6. Primary B5b IBC: Metastatic Breast Cancer

**Figure 5:**
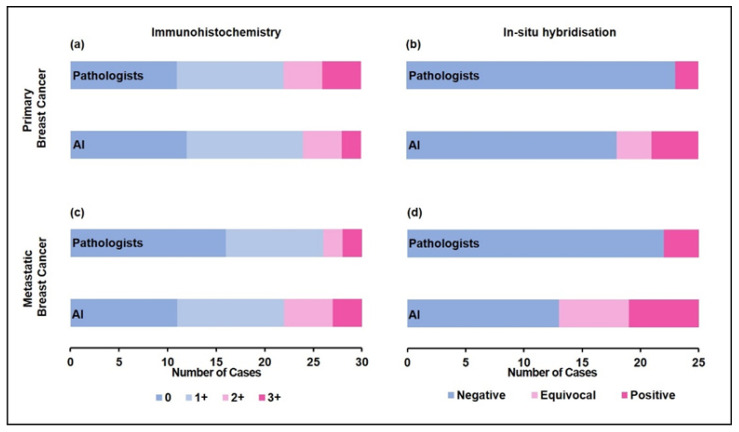
Primary breast cancer (**a**) IHC scores and (**b**) ISH status, and matched metastatic breast cancer (**c**) IHC scores and (**d**) ISH status assessed by the Pathologists versus AI. Equivocal ISH scores refer to groups 2, 3, and 4 in the 2018 ASCO/CAP guidelines.

For our cohort of 30 metastatic breast cancers, the accuracies of both the AI algorithms for IHC (Cohen’s κ 0.43, 95% CI 0.25–0.61) and ISH (Cohen’s κ 0.52, 95% CI 0.08–0.97) were moderate. The IHC algorithm performed slightly better on metastatic tumors (25/30 cases, Cohen’s κ 0.48, 95% CI 0.24–0.73) compared to their matched primary tumors (20/30 cases, Cohen’s κ 0.36, 95% CI 0.07–0.64) (Figure 5). Conversely, inter-observer agreement on IHC scoring between Pathologists was 4% higher on primary tumors compared to metastatic deposits (Fleiss’ κ primary 0.77 vs. metastatic 0.73). Of the 25 cases that underwent ISH assessment, three were positive according to the pathologist and six were positive according to AI. A conversion of HER2 status between the primary and metastatic lesions was observed in 1/30 cases, which was confirmed by both pathologists and AI with both IHC and ISH. A significant difference in the evaluation of metastatic tissue between the liver and lymph node sites was not observed.

### 3.7. Primary B5b IBC: Accuracy of AI-Assisted Digitalized Workflow

We incorporated both the HER2 IHC and ISH results in our assessment of how an AI-assisted, digitalized workflow compared to the manual assessment by pathologists. The ground truth HER2 status for all cases in our cohort was determined by consensus in the Pathologists’ opinions. In accordance with the ASCO/CAP guidelines, we only considered the ISH results for cases rated as 2+ by IHC. For cases evaluated as a score 0 or 1+ on IHC, we set a final HER2 negative status, and for cases evaluated as HER2 score 3+ by IHC, we set a final HER2 positive status. In total, 15% of cases were HER2 positive. When we compared the final HER2 status for each case as determined by the AI-assisted workflow versus their respective ground truths, we observed almost perfect agreement. The sensitivity and specificity for the IHC algorithm were 93.8% and 96.1% and for the ISH algorithm, 100% and 94.7%.

## 4. Discussion

This feasibility study demonstrates almost perfect agreement between a digitalized AI-assisted workflow and routine light microscopy for the determination of HER2 IHC and ISH status in our cohort of primary and metastatic breast carcinomas and according to the 2018 ASCO/CAP guidelines. While previous studies have reported on the performance of digitalizing individual steps in the workup of HER2 status, we have demonstrated, for the first time, the successful implementation of a complete digital AI-assisted workflow [17,21,22]. The overall HER2 positivity rate in our study cohort was 15%, which is consistent with the rates reported in the literature [23,24].

When considering the performance of the IHC algorithm independently, we observed a moderate concordance rate, which was similar to other algorithms described in the literature. Several studies assessing the performance of IHC-based AI algorithms have merged the IHC scores 0 and 1+ into a single ‘negative’ category, which markedly improved their overall concordance rates [17,21,25]. We also observed significant improvement when merging these groups, resulting in an almost perfect concordance (Cohen’s κ 0.59 vs. 0.85, *p* < 0.001 χ2 test). However, we acknowledge that the utility of this methodology is limited following the results of the DESTINY-Breast 04 trial concerning HER2 low tumors [10]. In their recent publication, Modi et al. concluded that trastuzumab deruxtecan prolongs progression-free and overall survival in patients with HER2 low tumors and called for a review of current HER2 diagnostic practices, given that “more than half of patients historically categorized as having HER2-negative breast cancer” could now exhibit improved treatment outcomes [10].

Extending upon these findings, preliminary results from the DAISY trial (due to be completed in 2025) are suggestive of antitumor activity by directed HER2 therapy in patients with an IHC HER2 score of 0 [11]. However, as observed in our study and by countless other groups, the HER2 IHC method and interpretation are plagued by low inter-rater agreement, particularly in the 0 and 1+ range [26]. Whilst the combined IHC/ISH method performed well for the detection of amplified and non-amplified HER2 tumors, it had shortcomings in the classification of HER2 low tumors. Alternative laboratory methods such as a combined protocol involving immunofluorescence and mass spectrometry may be capable of systematic and standardized quantification of drug-targetable HER2 amplification [26]. However, further studies stratifying molar HER2 quantities with response to therapy are required in order to understand if this approach is fit for purpose. In the midst of the uncertainty in defining the boundaries for the group with the best benefit–risk response profile to trastuzumab deruxtecan, it is crucial that the assessment of HER2 is as objective and standardized as possible.

The ISH-algorithm demonstrated substantial concordance with our ground truth. Whilst we noted a tendency for AI to provide higher HER2 counts and HER2/CEP17 ratios than paired manual slides, this did not have a significant impact on the overall diagnostic performance. Various reasons contributing to the higher counts included misinterpretation of the cell borders, misinterpretation of dark cytoplasmic staining secondary to cell shrinkage artefact as HER2 signals, and assigning higher counts to clusters. An advantage of the ISH algorithm is the heat-map overlay on the whole-slide image, which provides a continuous graded coloring system to delineate areas of normal and high HER2 signals/cell or HER2/CEP17 ratios, and thus aids the user in selecting an appropriate ROI. Due to the small size of HER2 and CEP17 signals, such insight is not possible through low-powered manual microscopy. The heatmap supports the user to distinguish tumor and non-tumor tissue, and may also facilitate the identification of clustered-type intratumoral heterogeneity in HER2 status [2,27]. This is important because emerging evidence suggests that different therapeutic approaches should be considered for HER2 heterogeneous breast carcinomas as they are less responsive to anti-HER2 therapies, display lower rates of pathologic complete response, and are associated with worse survival outcomes [27].

Before embarking on digitalization and incorporating artificial intelligence in diagnostic pathology, it is important to appreciate the potential impacts on pre-analytical, analytical and post-analytical procedures. For example, the IHC-algorithm used in this study was highly dependent on tight adherence to the vendor’s immunostaining protocol and could not be adapted to perform with the existing local laboratory practices. Therefore, for the purpose of this study, we deviated from our routine laboratory HER2 IHC staining protocol in order to match the vendor recommendation for their digital scanner and paired AI algorithm. In our experience, vendors are reluctant to adapt AI software for end-users on the basis that this may invalidate their CE-certification status. The changes in IHC staining protocols made by laboratories should undergo internal and external validation, and be suitable for both AI and manual interpretation.

Moreover, whilst we chose to analyze the performance of the AI-assisted DIA independently, without manual correction, its intended use is as a real-time diagnostic adjunct and it would therefore necessitate that all users have access and training. Interestingly, we noted a reduction in the accuracy of our Pathologists’ IHC scoring with the consensus scores after they had viewed the AI results (Cohen’s κ Pathologists 0.85–0.88 vs. AI-assisted Pathologists 0.61–0.71, t = 4.921, *p* < 0.05). This observation differs to other studies, which describe an AI-enhancing effect on diagnostic accuracy [28,29,30]. We also noted a reduction in the inter-observer agreement for AI-assisted Pathologists compared to Pathologists, however, this observation was not statistically significant in our cohort. It would be important to review and quantify the impact of AI on the existing reporting patterns as well as consider the time and cost-effectiveness prior to implementing new workflows [29].

A limitation of our work was the small sample size of our study cohort—whilst this was sufficient to demonstrate feasibility, it limited the statistical significance of our comparisons. According to recommendations from the CAP, FDA-approved and FDA-cleared HER2 IHC quantitative image analysis systems should be validated with 20 known positive and 20 known negative cases, and should show an agreement threshold of 90% for HER2 positive and 95% for HER2 negative samples [25]. Future studies combining the results from multiple institutions, and including resection specimens in addition to CNBs, would be beneficial to understand the impact of inter-laboratory and pre-analytical specimen processing and sampling on the performance of IHC and ISH methods [31].

In conclusion, this study demonstrates the feasibility of a combined IHC and ISH digitalized AI-assisted workflow for HER2 status determination in primary and metastatic breast cancer including the newly recognized group of HER2 low tumors.

## Figures and Tables

**Table 1:** Overview of the clinicopathological features for the preliminary (*n* = 495) and study (*n* = 97) cohorts. Tumor data for the study cohort pertain to the 67 primary tumors.

	Preliminary Cohort	Study Cohort
**No. of Cases**	495	97 (67 *)
**Average Patient Age, yrs**	61	60
**Range**	29–95	30–80
**Tumor Type, No. (%)**		
**Ductal**	394 (80)	55 (82)
**Lobular**	75 (15)	6 (9)
**Other**	26 (5)	6 (9)
**Tumor Grade, No. (%)**		
**1**	112 (23)	9 (14)
**2**	237 (48)	30 (45)
**3**	146 (29)	28 (42)
**ER positive, No. (%)**	421 (85)	58 (87)
**PR positive, No. (%)**	371 (75)	52 (78)
**HER2 positive, No. (%)**	59 (12)	10 (15)

* 67 were primary tumors, and clinicopathological data pertains only to primaries.

## Data Availability

The datasets generated and analyzed during the study are available from the corresponding author on reasonable request.

## Supplementary Materials

The following supporting information can be downloaded at: https://www.mdpi.com/article/10.3390/diagnostics13010168/s1, Table S1: IHC scores assigned by AI of 57 primary IBCs using laboratory staining compared to amended (vendor recommended) staining protocols.
