# Supplementary material for: Determining HER2 Status by Artificial Intelligence: An Investigation of Primary, Metastatic, and HER2 Low Breast Tumors
Source: Diagnostics (Basel). 2023 Jan 3;13(1):168. doi: 10.3390/diagnostics13010168 (PMC9818571; doi:10.3390/diagnostics13010168)
Supplement: Supplementary file 1 [file diagnostics-13-00168-s001.zip › diagnostics-2079471-supplementary.pdf]

Laboratory Staining Protocol	IHC Score	Amended Staining Protocol
2	0	25
20	1+	16
25	2+	10
10	3+	6

Supplement Table S1: IHC scores assigned by AI of 57 primary IBCs using laboratory staining compared to amended (vendor recommended) staining protocols.
